# SPME-GC–MS combined with chemometrics to assess the impact of fermentation time on the components, flavor, and function of Laoxianghuang

**DOI:** 10.3389/fnut.2022.915776

**Published:** 2022-08-02

**Authors:** Liu Yaqun, Liu Hanxu, Lin Wanling, Xue Yingzhu, Liu Mouquan, Zheng Yuzhong, Hu Lei, Yang Yingkai, Chen Yidong

**Affiliations:** ^1^School of Food Engineering and Biotechnology, Hanshan Normal University, Chaozhou, China; ^2^Guangdong Provincial Key Laboratory of Functional Substances in Medicinal Edible Resources and Healthcare Products, Chaozhou, China; ^3^Chaozhou Branch of Chemistry and Chemical Engineering Guangdong Laboratory (Hanjiang Laboratory), Chaozhou, China; ^4^Guangdong Jigong Healthy Food Co., Ltd, Chaozhou, China

**Keywords:** Laoxianghuang, SPME-GC-MS, fermentation time, volatile components, flavor, function

## Abstract

Laoxianghuang, fermented from *Citrus medica* L. var. *Sarcodactylis Swingle* of the *Rutaceae* family, is a medicinal food. The volatiles of Laoxianghuang fermented in different years were obtained by solid-phase microextraction combined with gas chromatography–mass spectrometry (SPME-GC–MS). Meanwhile, the evolution of its component-flavor function during the fermentation process was explored in depth by combining chemometrics and performance analyses. To extract the volatile compounds from Laoxianghuang, the fiber coating, extraction time, and desorption temperature were optimized in terms of the number and area of peaks. A polydimethylsiloxane/divinylbenzene (PDMS/DVB) with a thickness of 65 μm fiber, extraction time of 30 min, and desorption temperature of 200 °C were shown to be the optimal conditions. There were 42, 44, 52, 53, 53, and 52 volatiles identified in the 3rd, 5th, 8th, 10th, 15th, and 20th years of fermentation of Laoxianghuang, respectively. The relative contents were 97.87%, 98.50%, 98.77%, 98.85%, 99.08%, and 98.36%, respectively. Terpenes (mainly limonene, γ-terpinene and cymene) displayed the highest relative content and were positively correlated with the year of fermentation, followed by alcohols (mainly α-terpineol, β-terpinenol, and γ-terpineol), ketones (mainly cyclohexanone, D(+)-carvone and β-ionone), aldehydes (2-furaldehyde, 5-methylfurfural, and 1-nonanal), phenols (thymol, chlorothymol, and eugenol), esters (bornyl formate, citronellyl acetate, and neryl acetate), and ethers (n-octyl ether and anethole). Principal component analysis (PCA) and hierarchical cluster analysis (HCA) showed a closer relationship between the composition of Laoxianghuang with similar fermentation years of the same gradient (3rd-5th, 8th-10th, and 15th-20th). Partial least squares discriminant analysis (PLS-DA) VIP scores and PCA-biplot showed that α-terpineol, γ-terpinene, cymene, and limonene were the differential candidate biomarkers. Flavor analysis revealed that Laoxianghuang exhibited wood odor from the 3rd to the 10th year of fermentation, while herb odor appeared in the 15th and the 20th year. This study analyzed the changing pattern of the flavor and function of Laoxianghuang through the evolution of the composition, which provided a theoretical basis for further research on subsequent fermentation.

## Introduction

People's dietary requirements have been increasing in recent years as their living levels have improved. The development of novel natural health foods has become a new trend, particularly novel foods made from medicine and food homologous (MFH) dual-use resources, which are becoming increasingly popular among consumers (1). Food fermentation is an ancient food processing method that is regarded as one of the most fundamental, natural, and useful procedures. Traditional fermented foods such as grains (2), legumes (3), and dairy products (4) have been reported, but there have been few reports of fermented MFH, which is a combination of medicine-food fermentation that enhances nutrition and efficacy while allowing its medicinal properties to play a unique role.

Laoxianghuang is an intangible cultural heritage unique to the Chaoshan region of Guangdong Province, China. It is a healthcare medicinal food made from the fermented fruit of *Citrus medica* L. var. *Sarcodactylis Swingle* (also known as Finger Citron, *Rutaceae* family MFH) and is used not only as a traditional medicinal plant but also as a delicious food. *Citrus medica* L. var. *Sarcodactylis Swingle* needs to go through a complex pretreatment process of salting, desalting, sugaring, cooking and drying, and fermentation for more than 3 years to obtain Laoxianghuang (5). During a series of complex fermentation processes, the external morphological characteristics and internal tissue chemical composition of the fruit undergo significant changes. Previous studies have shown that long-fermented Laoxianghuang reduces harmful microorganisms and increases *Bifidobacterium* and *Lactobacillus* compared to fresh fruit (6). According to the 2015 edition of the Chinese Pharmacopeia, it has effects in soothing the liver and regulating gas, relieving pain in the stomach, eliminating dampness, and resolving phlegm (7). Similar to MFH food *Citri Reticulatae Pericarpium* (also in the family *Rutaceae*), it is also traditionally believed that the longer Laoxianghuang is fermented, the better the quality is, that is, “the older the better” (8). However, its current large-scale production is limited, relying mainly on small-scale production, with few standardized production companies. Therefore, the fermentation procedure of Laoxianghuang is based on individual experience and lacks standardized process techniques and proper monitoring indicators. The quality of products obtained through fermentation varies greatly, making it difficult to establish uniform quality standards and testing methods to ensure safety and efficacy.

*Citrus medica* L. var. *Sarcodactylis Swingle* is often used as a perfume and food flavoring additive because of its delicate flavor. The flavor in food is caused by a variety of fragrance-presenting volatiles. Previous reports on the flavor-related determination of *Citrus medica* L. var. *Sarcodactylis Swingle* mostly used GC for isolation and detection (9). GC–MS showed that the volatile components of the original fruit were mainly limonene and were more pronounced in the maturation stage with α-thujone, 3-carene, α-pinene, β-pinene, γ-terpinene, monoterpene hydrocarbons, and ketones (10). Although *Citrus medica* L. var. *Sarcodactylis Swingle* is added to beverages, pastries, or candies because of its unique aroma, its taste is too bitter and spicy, resulting in only trace amounts of flavoring. Therefore, *Citrus medica* L. var. *Sarcodactylis Swingle* is generally used for fermentation, while the Laoxianghuang formed after fermentation has a stronger and sweeter flavor that can be consumed directly and is commonly used locally to make water for drinking. Fermentation not only improved the flavor of *Citrus medica* L. var. *Sarcodactylis Swingle* but also maintained the relevant medicinal effects. The purpose of this study was to reveal the flavor mechanism and further analyze the flavor formation and quality characteristics during the fermentation process through the determination of volatile components.

SPME-GC–MS has become increasingly popular in recent years for the identification, authentication, characterization, and classification of complex natural samples such as traditional Chinese medicine (TCM) and food (11, 12). It is a promising tool for the qualitative analysis of ingredients and can be effectively used for product quality control. SPME combines sampling, analyte separation, and enrichment to pretreat volatile compounds in complex matrices (13), is the most preferred means of collection, and has been extensively explored in wine (14), meat (15), fruit (16), and TCM (11). The application of SPME-GC–MS in fermentation products has been increasingly expanded in recent years; for instance, esters and alcohols had the most abundance on the 98th day of fermentation of grains into Baijiu (17). Acetic acid was positively correlated with fermentation time after 96 h of *Coffea arabica* fermentation, while 2-methylpyrazine, 2-furanmethanol, 2,6-dimethylpyrazine, and 5-methylfurfural showed the opposite trend (18). After black currant (*Ribes nigrum*) was fermented for 12 days, the contents of esters, higher alcohols, and fatty acids increased significantly (19). The dynamic changes in volatile components during the fermentation of ferments obtained by SPME-GC–MS can reveal the evolution of the kinetics, physicochemical properties, flavor, and so on, which provides a strategy to further improve the characterization of ferments. GC–MS can determine volatile compounds with high precision, and combined with SPME, GC–MS can determine the changes in volatile components of Laoxianghuang at different fermentation times. It is also noteworthy that the chemical characteristics of Laoxianghuang are variable and complex with different fermentation times. The analyzed data were processed by chemometrics, which is commonly used as an exploratory mathematical analysis tool well suited for food identification, to make the results clearer. Principal component analysis (PCA), partial least squares discriminant analysis (PLS-DA), and hierarchical cluster analysis (HCA) are most often used to process and extract potentially useful information from multivariate datasets (20).

In this study, SPME-GC–MS combined with chemometrics methods was used to effectively evaluate the dynamic patterns of composition, flavor, and function of Laoxianghuang at different fermentation stages, with the aim of providing new ideas for subsequent related fermentations and making useful contributions to further in-depth studies of Laoxianghuang.

## Materials and methods

### Materials

Different fermentation years of Laoxianghuang (3rd, 5th, 8th, 10th, 15th, and 20th years) were supported by Guangdong Jigong Healthy Food Co., Ltd. (Chaozhou, Guangdong Province, China). Laoxianghuang was sampled by randomly taking six samples of the same fermentation year from the fermenter, chopped, and mixed them. Then, the samples were dried at 40 °C for 36 h until the moisture content was <15%, crushed into a powder with a particle size of 30 mesh, and frozen at −20 °C until further use.

### Optimization of SPME analysis

A total of 1.0 g of sample and 3 mL of saturated NaCl solution were mixed, sealed in a 20 ml headspace vial, and pre-equilibrated for 15 min at 50 °C in a thermostatic bath with a vial capped using a silicon septum. Selection of different fibers: polydimethylsiloxane (PDMS) with a thickness of 100 μm, divinylbenzene/carboxen/polydimethylsiloxane (DVB/CAR/PDMS) with a thickness of 50/30 μm, and polydimethylsiloxane/divinylbenzene (PDMS/DVB) with a thickness of 65 μm (Supelco, USA) were first manually inserted into the sample vial that had been incubated in a dry heat block for 30 min at 50 °C. After extraction, the fibers were immediately injected into the GC–MS injection port for desorption at 200 °C for 5 min. Selection of extraction time: The optimal fiber was used, and the samples were incubated in a dry heat block at 50 °C for 15, 30, and 45 min. The subsequent experimental operations were performed in the same way as the optimized fiber. Selection of desorption temperature: Based on the optimal fiber and extraction time described earlier, samples were injected into the GC–MS inlet immediately after processing and desorbed for 5 min at 150 °C, 200 °C, and 250 °C in splitless mode. In all the analyses, the same batch of Laoxianghuang from the same year was used for a single variable test under the same conditions of other factors. The optimal fiber, extraction time, and desorption temperature were selected by comparing the total peak area and the number of peaks in the chromatogram.

### GC–MS conditions

The volatile compounds of Laoxianghuang were detected by a 7890A gas chromatograph connected to a 5977A mass spectrometer (Agilent Technologies, Santa Clara, CA); separation was performed using an HP-5MS column (30 m × 0.25 mm i.d. with 0.25 μm film thickness). The injection was performed in splitless mode. Helium was used as the carrier gas at a constant flow rate of 1.0 mL/min. The oven temperature was programmed as follows: initial 50 °C for 2 min, increased to 85 °C at a rate of 10 °C/min and held for 10 min, then raised to 120 °C at 15 °C/min and held for 2 min, and finally increased to 145 °C at 2 °C/min and held for 5 min. The injector temperature was 200 °C, and the carrier gas was helium with a flow rate of 1.0 mL/min. The detector was set in electron impact (EI) ionization mode at 70 eV in full scan mode with a mass/charge ratio (m/z) range from 45 to 450.

The chromatogram peak obtained by GC–MS was searched by the National Institute of Standards and Technology (NIST14.L) standard mass spectrometry library of the chemical workstation, and the unidentified components were qualitatively analyzed. The relative percentage content of each compound in the volatile components of Laoxianghuang was calculated relatively quantitatively by the peak area normalization method (5).

### Statistical analysis

Data from three independent replicates of GC–MS were processed with SPSS statistical software (Version 21.0, SPSS Inc., Chicago, IL, USA) and statistically analyzed by one-way analysis of variance (ANOVA). Differences were considered significant at p < 0.05, and the results are expressed as the mean values ± standard deviation (SD). PCA, PLS-DA, and HCA were performed with MetaboAnalyst 5.0 (http://www.metaboanalyst.ca) (21). The flavor was queried from Flavornet (http://www.flavornet.org/flavornet.html) (22), Linear Retention Indices (LRI), and Odor Database (http://www.odour.org.uk/) (23). Compound-related efficacy was obtained from the Comparative Toxicogenomics Database (CTD, http://ctdbase.org/) (24) and the Traditional Chinese Medicine Systems Pharmacology Database and Analysis Platform (TCMSP, https://old.tcmsp-e.com/tcmsp.php) (25). Flavor and function network diagrams were constructed by Cytoscape software (Version 3.9.1, https://cytoscape.org/) (26).

## Results

### Optimization and method validation of SPME

**Figure 1:**
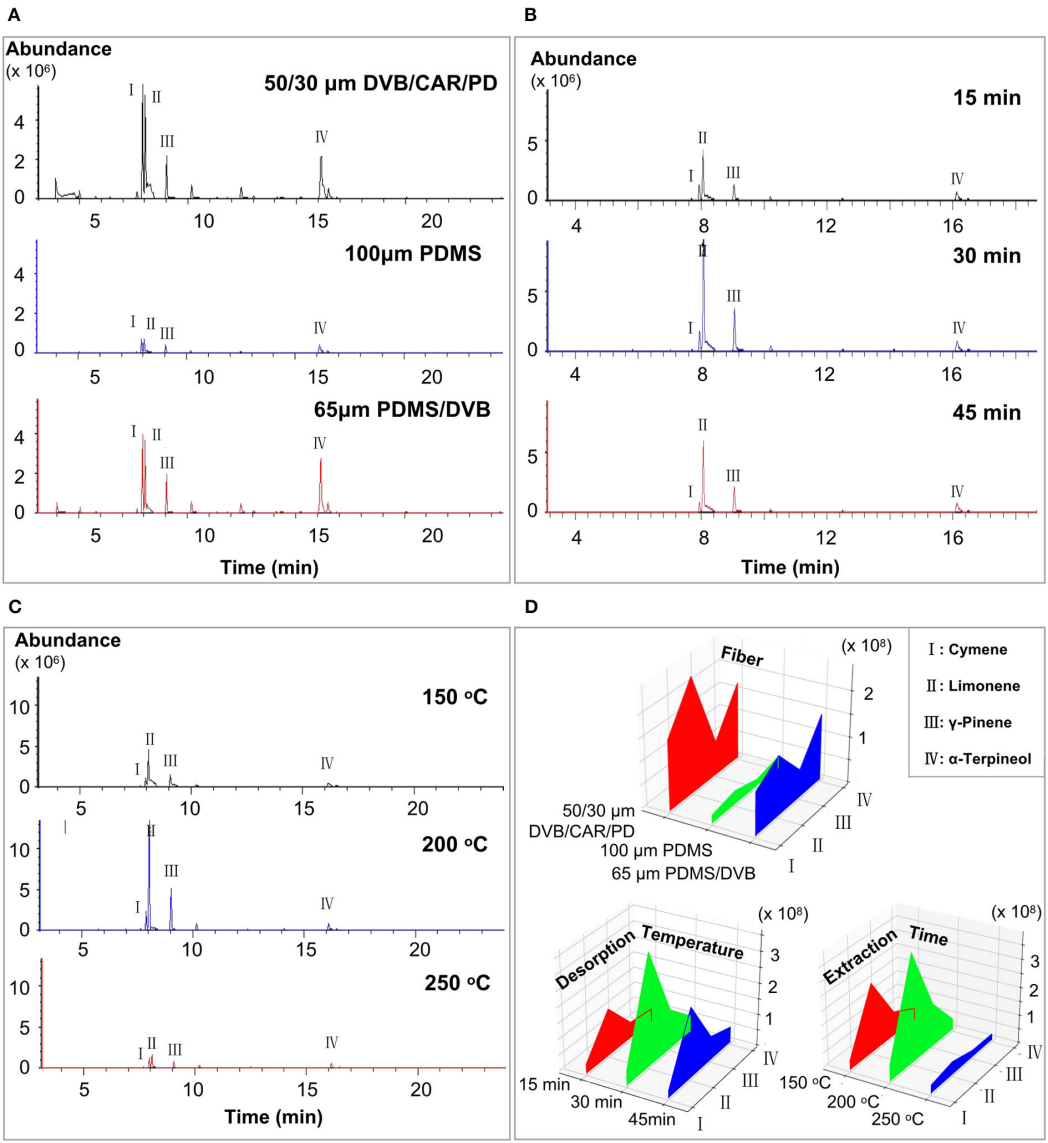
Chromatograms obtained from different optimized conditions and peak areas of the four main reference components. **(A)** Chromatograms obtained from 100 μm PDMS, 50/30 μm DVB/CAR/PDMS, and 65 μm PDMS/DVB fibers. **(B)** Chromatograms obtained at extraction times of 15 min, 30 min, and 45 min. **(C)** Chromatograms obtained at desorption temperatures of 150 °C, 200 °C, and 250 °C. **(D)** Peak areas of cymene, limonene, γ-pinene, and α-terpineol detected at different fibers, extraction times, and desorption temperatures.

The results of a previous study indicated that cymene, limonene, γ-pinene, and α-terpineol were relatively more abundant in Laoxianghuang (5); therefore, these four substances were used as references in the study of the effects of fiber, extraction time, and desorption temperature on SPME. Comparing the extraction results of the samples by 100 μm PDMS, 50/30 μm DVB/CAR/PDMS, and 65 μm PDMS/DVB, the 50/30 μm DVB/CAR/PDMS detected the highest number of peaks with 61 volatile compounds identified, while 65 μm PDMS/DVB and 100 μm PDMS were listed in descending order with 55 and 53 compounds identified, respectively. However, the chromatogram of 50/30 μm DVB/CAR/PDMS showed serious peak trailing and distortion compared with the 65 μm PDMS/DVB (Figures 1A,D). Thus, the fiber with a 65 μm PDMS/DVB coating was selected as the best choice for the identification of Laoxianghuang volatile compounds.

There was little difference in the number of peaks in the chromatograms at the extraction times of 15 min, 30 min, and 45 min. The extraction of the main volatile components was completed and reached adsorption equilibrium (Figure 1B). Remarkably, the peak areas of the four reference substances were significantly larger at an extraction time of 30 min than at 15 min and 45 min (Figure 1D). This may be because continuing to increase the extraction time after the fiber has reached extraction saturation will result in competing for adsorption of volatile components in the sample vial, thus causing a decrease in the peak area of the major volatiles. In terms of desorption temperature, the peak numbers and areas of volatile major components were lower at 150 °C and 250 °C compared to the desorption temperature of 200 °C (Figure 1C), while the peak areas of the reference substances were higher and clearer at 200 °C (Figure 1D). Therefore, the optimal extraction equilibrium time and desorption temperature for the volatile compounds of Laoxianghuang in this study were confirmed to be 30 min and 200 °C, respectively.

### Volatile compound profile of the Laoxianghuang

**Figure 2:**
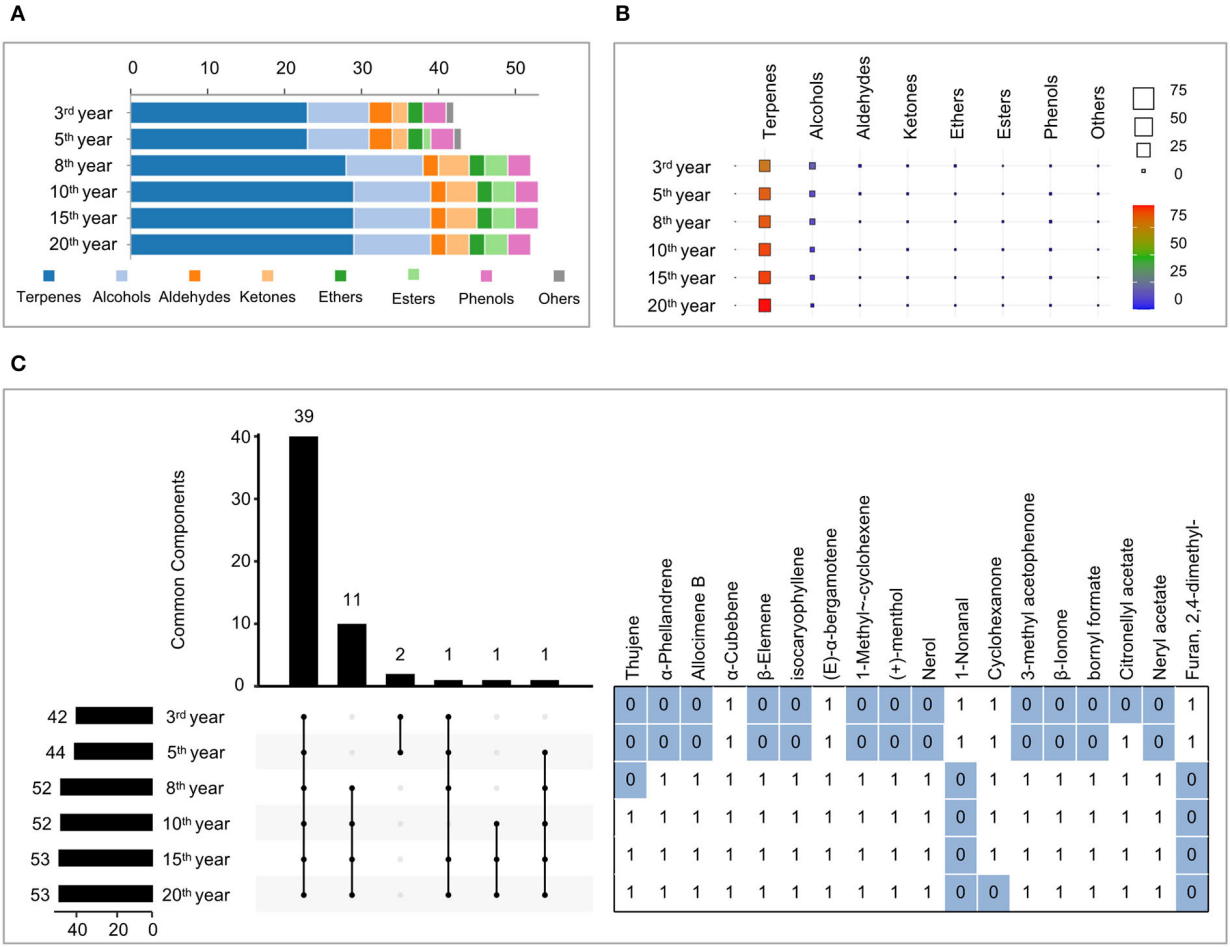
Composition, content, and commonality of volatile compounds of Laoxianghuang in different fermentation years. **(A)** A number of terpenes, alcohols, aldehydes, ketones, ethers, esters, phenols, and others in Laoxianghuang. **(B)** Total relative contents of volatiles in Laoxianghuang for the eight chemical categories. **(C)** UpSet plot showing the co-occurrence of volatile components in Laoxianghuang at different fermentation times. The total size of each set is represented by the bar plot on the left. Each possible intersection is represented by the bottom plot, and their occurrence is shown on the top bar plot. Detailed table with the frequency of presence of volatile components: presence is indicated by 1, absence by 0. Only components with differences are listed. The other 39 components not listed are present at different times (i.e., all are indicated by 1); these include α-pinene, camphene, β-pinene, myrcene, terpinolene, cymene, limonene, ocimene, γ-terpinene, α-cubebene, (E)-α-bergamotene, β-caryophyllene, α-humulene, (E)-β-farnesene, β-santalene, γ-muurolene, germacrene B, isoledene, α-muurolene, (Z)-α-bisabolene, β-bisabolene, γ-cadinene, δ-cadinene, fenchol, 1-terpinenol, β-terpinenol, dl-isoborneol, borneol, terpinen-4-ol, α-terpineol, γ-terpineol, 2-furaldehyde, 5-methylfurfural, D (+)-carvone, N-octyl ether, anethole, thymol, chlorothymol, and eugenol.

GC–MS analysis was performed under the optimized SPME experimental parameters and characterized the volatility of Laoxianghuang at different fermentation times. A total of 55 compounds in Laoxianghuang were identified by the mass spectra matched to NIST14.L with a match quality score ≥ 80. Based on their chemical structures and properties, the identified compounds were classified into eight chemical categories. Terpenes (29) were the numerous volatile compounds, followed by alcohols (10), ketones (4), aldehydes (3), esters (3), phenols (3), ethers (2), and others (1) (Supplementary Table 1 and Figures 2B, **5A**).

The number and categories of identified volatile compounds in Laoxianghuang, as well as their relative contents, varied over the different fermentation times. In total, 42 and 44 volatile compounds were identified at the 3rd and 5th years of fermentation, which were significantly less than the 52, 53, 53, and 52 at the 8th, 10th, 15th, and 20th years. Among them, the number of terpenes, alcohols, ketones, and esters increased with increasing fermentation time, while the number of aldehydes, ethers, and phenols did not change significantly (Figure 2A). It is noteworthy that the compound Furan, 2,4-dimethyl- appeared only in the 3rd and 5th years of initial fermentation (**Figure 5A**).

Terpenes were the main volatiles of Laoxianghuang with relative contents ranging from 76.18 to 94.99%, following the same trend as its quantity and positively correlated with the fermentation time. In contrast, the relative content of alcohols showed an inverse phenomenon; although the number increased, the relative content decreased with the year of fermentation from 19.4% in the 3rd year to 2.84% in the 20th year. Aldehydes, ketones, and ethers showed the same trend as alcohols, all showing high levels at the beginning of fermentation. However, esters and phenols showed the highest dynamic changes in the 8th and 10th years of fermentation.

Due to the diversity of volatile compounds, the UpSet plot was used to group data points that had many identical values in different features together and to find the largest intersecting sets. All fermentation years of Laoxianghuang contained the same 39 compounds. Nerol and furan are absent from the eighth year of fermentation for Laoxianghuang, although the variety of volatile components increases significantly compared to the third and fifth years of fermentation (Figure 2C).

### Multivariate statistical analysis of Laoxianghuang

**Figure 3:**
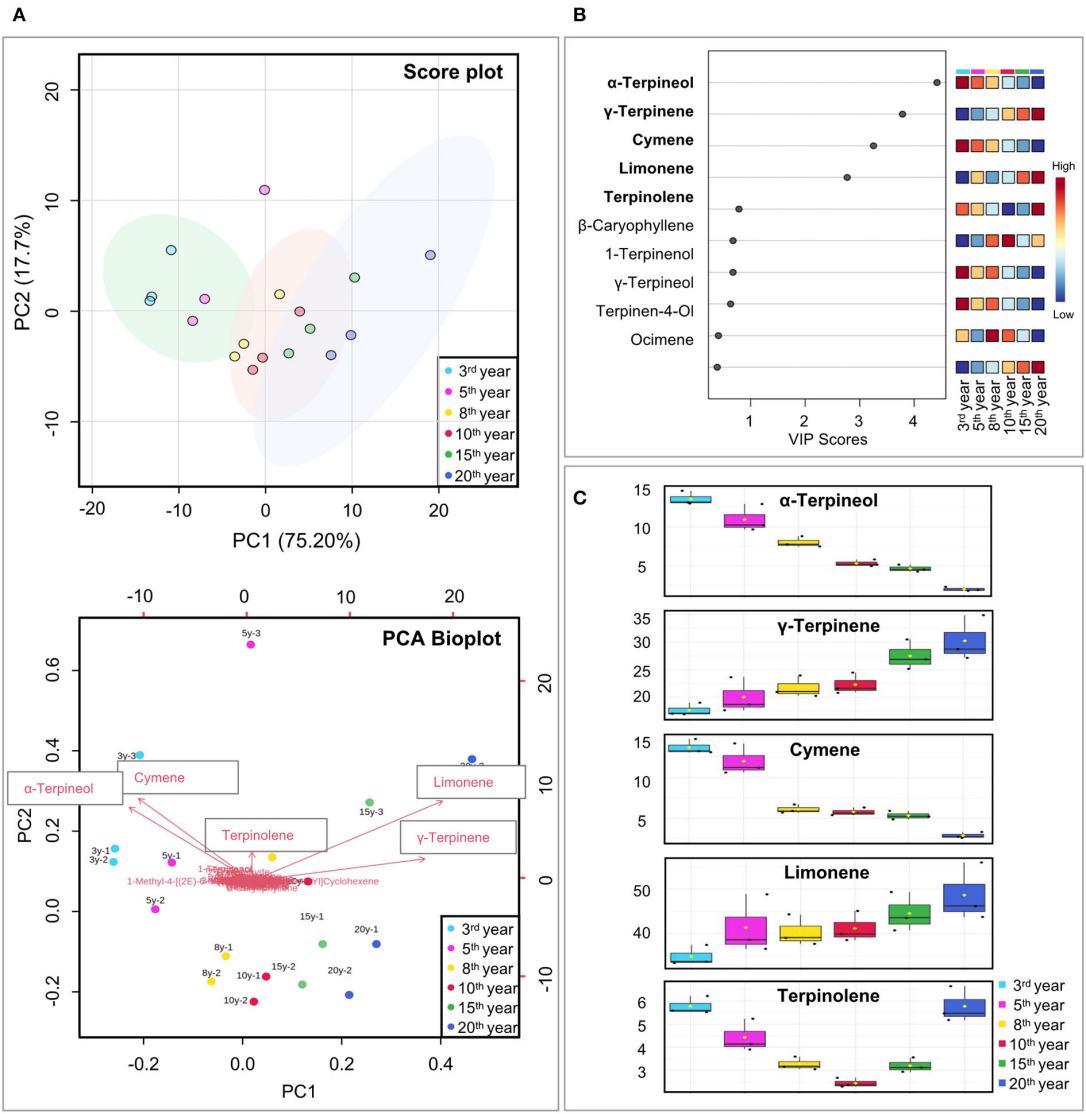
Selection of volatile markers for Laoxianghuang in six different fermentation years. **(A)** 2D score plot of PCA for PC1 and PC2 (above) and their biplot (below, including the position of each sample for PC1 and PC2 and how the variables map to these samples). Sample replicates are shown by the small circles of the same color. **(B)** VIP of PLS-DA score plots. A VIP score greater than 1 of the compound was considered to indicate differential volatility. **(C)** Relative content of candidate markers selected by PCA and PLS-DA.

The unsupervised PCA clustering method was applied to all volatile compounds, and the results showed good repeatability for each fermentation year, confirming the feasibility of optimizing the SPME-GC–MS method. The PCA score plot showed that the contributions of PC1 and PC2 were 75.2 and 17.7%, respectively, with a cumulative contribution of 92.9%, which could reflect most of the information of the samples. Six different fermentation years were clustered into three subgroups, with the 3rd and 5th years (highlighted with a green circle), the 8th and 10th years (highlighted with a pink circle), and the 15th and 20th years (highlighted with a blue circle). The presence of specific components was explored using PCA biplot visualization. Each arrow in the plot corresponds to a component. The longer the arrow is, the greater the influence and importance of the corresponding substance for that PC. The results showed that five substances limonene, α-terpineol, cymene, γ-terpinene, and terpinolene were more remarkable compared to the other components (Figure 3A).

The variable importance in projection (VIP) method was used to calculate and identify the characteristic volatile metabolites in the PLS-DA model. When the VIP value was >1, the variable was considered to have an important function in the PLS-DA discriminant process. In the VIP score plot, the VIP values of α-terpineol, γ-terpinene, cymene, and limonene were >1.0 in descending order, indicating that these volatile compounds may play a role as potential differential volatiles in Laoxianghuang of different fermentation years (Figures 3B,C).

**Figure 4:**
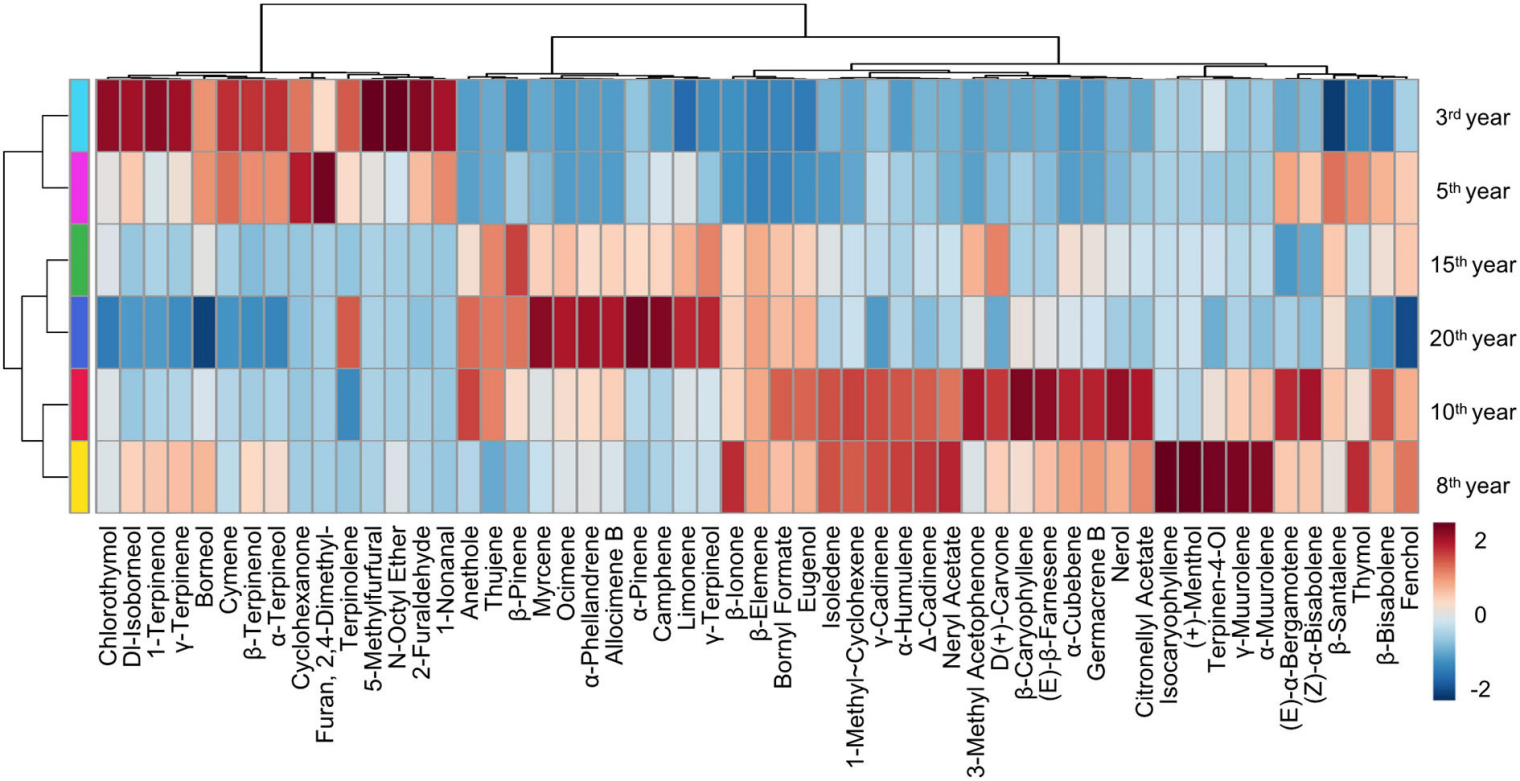
HCA of Laoxianghuang based on the normalized quantities of the identified volatiles.

To present more intuitive and effective information about the composition of Laoxianghuang in different fermentation years, a heatmap with hierarchical clustering was used. The six different fermentation years of Laoxianghuang in the heatmap were divided into three main categories, which were consistent with the results of PCA. The first cluster contains the 3rd and 5th years of fermentation with relatively high contents of alcohols, ethers, and aldehydes; the second cluster contains the 15th and 20th years with high contents of alkenes; and the third cluster contains the 8th and 10th years with relatively high contents of not only all of the abovementioned types of compounds but also phenols and ketones. In addition, compared to the 3rd year of fermentation, the type and content of volatile components of Laoxianghuang changed significantly with the later years of fermentation, and the 5th and 8th years were considered to be the transitional years of change (Figure 4).

### Influence of fermentation on the flavor characteristics of Laoxianghuang

**Figure 5:**
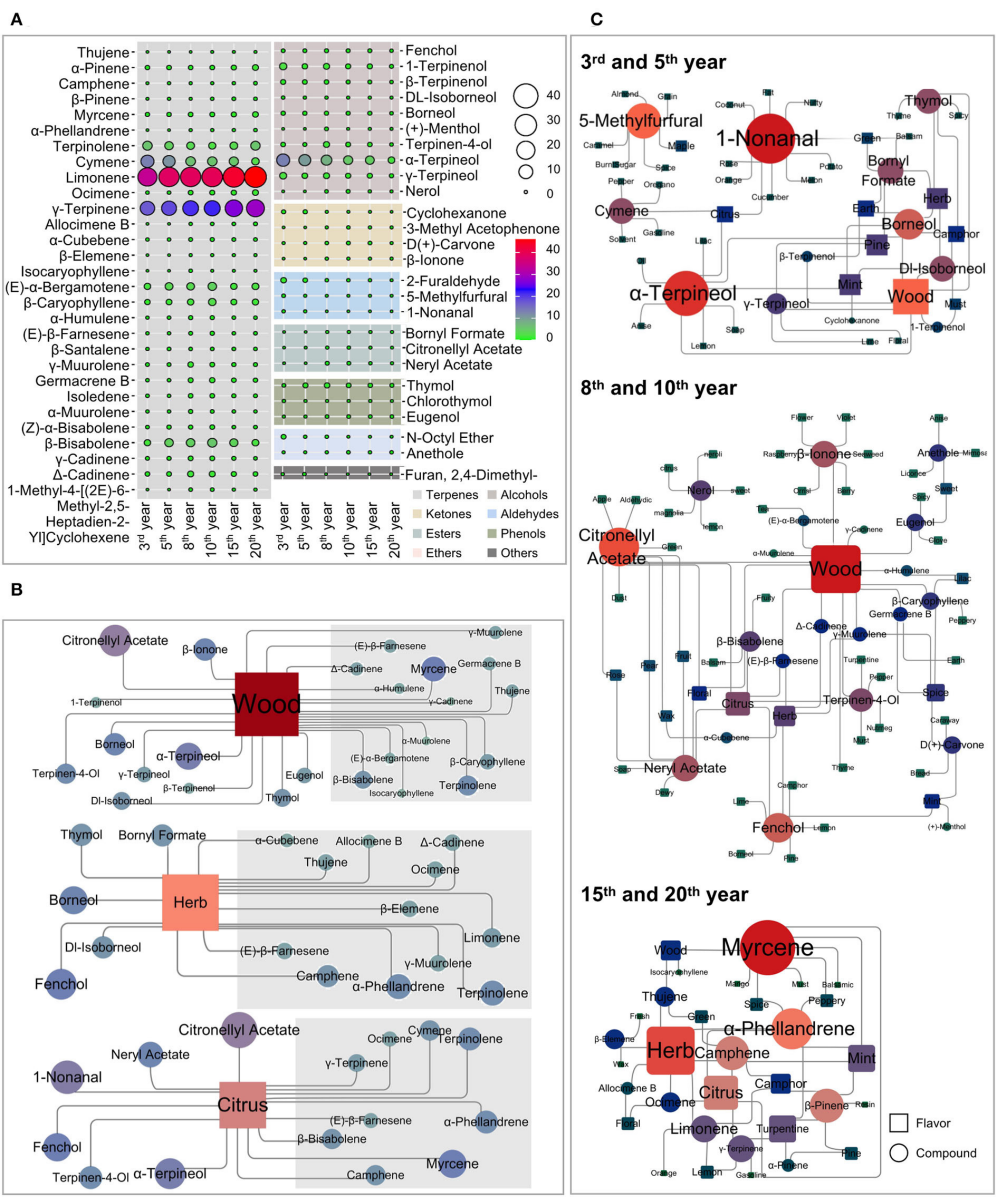
Flavor correlation diagram of volatile compounds of Laoxianghuang. **(A)** Comparison of the composition of fermented Laoxianghuang from different years. **(B)** Network diagram of the characteristic flavor and related volatiles of Laoxianghuang. **(C)** Network diagram of the relationship between the flavor and composition of Laoxianghuang in different fermentation years. Square indicates flavor, circle indicates volatiles, and the size indicates its correlation. The redder the color, the more correlates there are; bluercolor indicates the opposite.

Based on the known flavor descriptors for identifying volatiles, the most characteristic flavor attributes in Laoxianghuang from different fermentation years could be predicted (Supplementary Table 1). The most frequent descriptors included wood (25), herb (17), citrus (15), and mint (11). Other important descriptors for these samples were also given for pine (8), camphor (7), spice (7), floral (7), green (5), turpentine (5), and lemon (5) (the number in parentheses is the amount of the corresponding fragrance volatiles). There were significant differences in the flavor attributes of the six different fermentation years, which means that their flavor qualities were highly variable. Specifically, in the 3rd and 5th years of fermentation, Laoxianghuang had a strong wood odor, followed by a higher intensity of herb, mint, pine, and citrus. The 8th and 10th years of fermentation were found to be dominated by the wood odor as well; however, they were then accompanied by more pronounced odors of citrus, herb, spice, and floral. After the 15th and 20th years of fermentation, the main volatiles of Laoxianghuang showed herb odor, followed by citrus, turpentine, mint, and wood (Figure 5C).

The wood, herb, and citrus odors were not only present throughout the fermentation process but also had a relatively high amount and content of their corresponding volatiles, which were mostly focused in the terpene group (gray areas in the figure). In particular, the content of terpenes far exceeded the odor threshold and was characteristic of all three odors. Volatile substances associated with wood odors also include the alcohol group. In addition to terpinolene, (E)-α-bergamotene, β-bisabolene, and α-terpineol are also volatiles associated with this characteristic and have high relative contents. The volatiles with relatively high contents of herb and citrus odors included limonene and cymene, γ-terpinene, β-bisabolene, and α-terpineol, respectively (Figure 5B).

### Influence of fermentation on the medicinal function of Laoxianghuang

**Figure 6:**
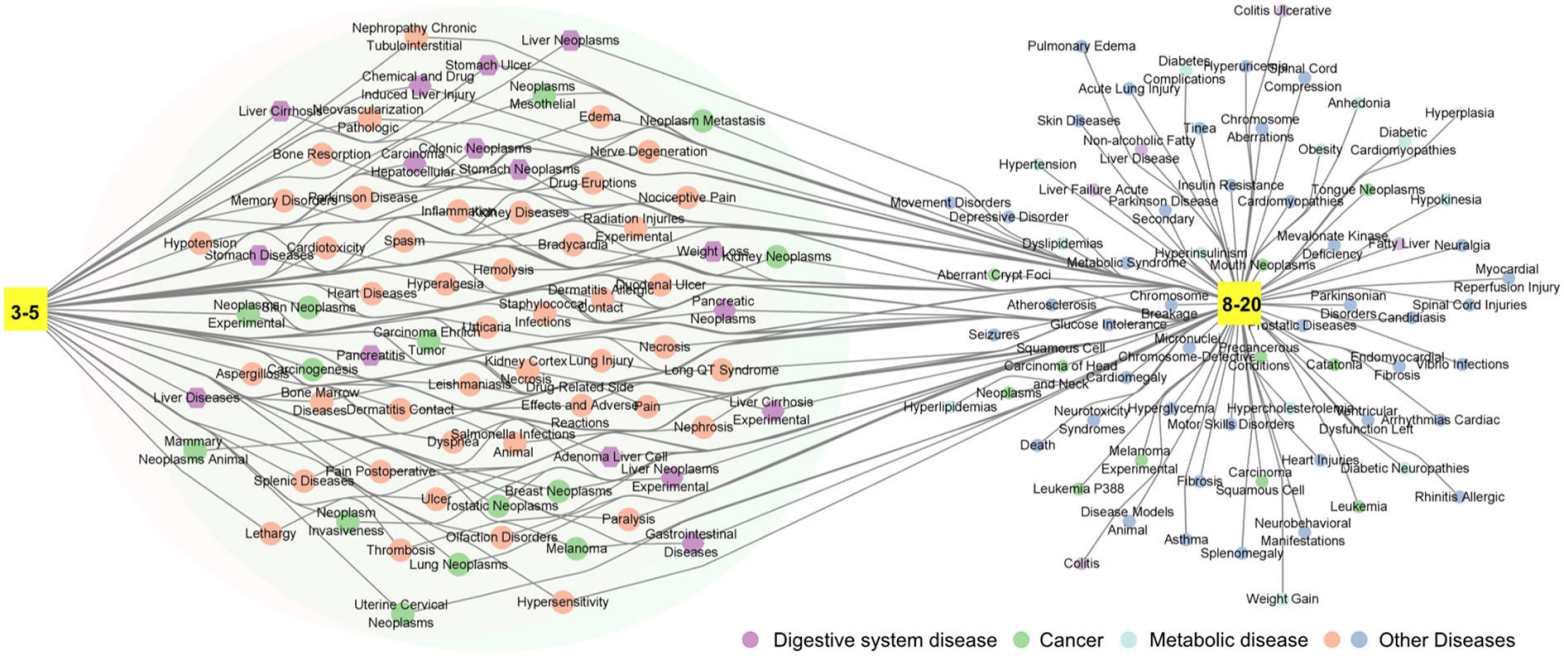
Network diagram of the medicinal functions of Laoxianghuang. 3–5: third and fifth years of fermentation; 8–20: eighth, tenth, fifteenth, and twentieth years of fermentation; the area covered by two large circles: common medicinal functions.

To investigate the effect of fermentation time on Laoxianghuang, the efficacy corresponding to the active ingredients detected in different years was searched and analyzed in the CTD and TCMSP databases. It was clear that the medicinal functions of Laoxianghuang fermented for more than 8 years encapsulated those of the lower years. This result is also reflected in the variety of its compounds, with significantly fewer components from the 3rd and 5th years of fermentation than from the 8th to 20th years. After fermentation for 3 to 20 years, the components of Laoxianghuang have all shown pathophysiological correlations with digestive system diseases and cancer. With the increase in fermentation years, that is, Laoxianghuang fermented for 8 to 20 years showed interactions with metabolic diseases such as obesity, hypertension, and diabetes, in addition to the abovementioned functions (Figure 6).

## Discussion

In recent years, the SPME-GC–MS technique has been increasingly applied to the determination of volatile substances in edible plants of the *Rutaceae* family (27–29), and this technique has also been applied to the detection of components in the fermentation process of *Citrus medica* L. var. *Sarcodactylis Swingle* (29). A series of characterization variations caused by its determination of components during the fermentation of wine, coffee, and other fermented products have also been reported (17–19). On this basis, this study detected the changes in volatile substances of Laoxianghuang (i.e., ferments of *Citrus medica* L. var. *Sarcodactylis Swingle*) in different fermentation years using SPME-GC–MS and further explored the patterns of flavor and functional evolution.

The effects of different fibers, extraction times, and desorption temperatures on the resolution of volatile components were initially investigated. After comparing the separation effect, compound categories, and other factors, the 65 μm PDMS/DVB fiber, extraction time of 30 min, and desorption temperature of 200 °C were finally selected as the optimal conditions for further analysis. This result differs from the previously reported detection of fermentation components of *Citrus medica* L. var. *Sarcodactylis Swingle* using CAR/PDMS fibers (29), which may be related to the change in volatiles caused by the fermentation time.

The volatile compounds were identified by GC–MS under the above-optimized conditions for different fermentation years of Laoxianghuang. The relative contents of terpenes were the highest, followed by alcohols, aldehydes, phenols, ketones, ethers, and esters. This is in common with other *Rutaceae*, especially citrus edibles such as Yuzu Jeune, Oni Yuzu, Bizzarria orange, Florence cedar, and other different varieties of citrus, which also possess the highest relative content of terpenes (29). Meanwhile, limonene and γ-terpinene were the main terpenoids present, followed by cymene and terpinolene, and this trend was also consistent in Laoxianghuang, Finger citron (30), and citrus (31). However, the contents of β-pinene and α-terpineol showed an opposite situation in citrus and Laoxianghuang, with α-terpineol more enriched in Laoxianghuang and β-pinene more abundant in citrus. Interestingly, the medicinal *Rutaceae* plant *Citrus aurantium* L. var. *amara Engl* has a high content of α-terpineol but a low content of other terpenes (32). Other fruits, such as grape (33) and Omija (34), also showed an increasing trend in α-terpineol content during fermentation. Terpenes are known to play a positive role mainly in flavor, while α-terpinol exhibits antitumor, hypolipidemic, and antibacterial effects (35, 36). According to the relative contents of the main compounds, we speculate that Laoxianghuang not only retained the characteristic flavor of *Rutaceae* during the fermentation process but also stimulated possible medicinal active ingredients.

Volatile components obtained from Laoxianghuang of different fermentation years were evaluated and analyzed using multiple chemometric methods, including PCA, PLS-DA, and HCA. The results clearly showed that the components of Laoxianghuang were clustered into three groups according to the fermentation years: 3–5 years, 8–10 years, and 15–20 years. This phenomenon is similar to the fermentation of grape wine (37), the aging of tea (38), and the storage of tangerine peel (39), where samples of the same fermentation or storage age shared some similarity and had the potential for clustering homogeneity. As with unfermented raw fruits (30) and other citrus (40), the VIP scores of PLS-DA were >1 for terpenes such as γ-terpinene, cymene, limonene, and terpinolene, which may be the signature characteristic compounds of such fruits. Unfortunately, α-terpineol was poorly reported in *Rutaceae*, while the VIP score is >1 in Laoxianghuang. This is identical to the medicinal plant *Ephedra sinica*, which is considered a pharmacologically important chemical marker of *Ephedra sinica* (41).

Because of the characteristic differences in the composition of Laoxianghuang in different fermentation years, the evolution pattern of its flavor and efficacy during the fermentation process was further investigated. By analyzing the flavor of each component, Laoxianghuang showed a preference for wood odor at the beginning of fermentation, with herb odor predominating in the later stages, and citrus odor was present throughout the fermentation process. It can be seen that the fermentation characteristics of Laoxianghuang are such that its flavor is not static but a continuous process of variation, and the time span of this change is measured by years. The fermentation characteristics of Pu-erh tea (42, 43) and Keemun black tea (44) also present the same trend, mainly expressed as floral and fruity flavors in the early stage of storage, woody flavors in the middle stage, and herbal flavors over 10 years.

The variation in composition caused by fermentation not only changed the flavor of Laoxianghuang but also diversified its functions. With prolonged fermentation years, the functions of Laoxianghuang have become more abundant, with pharmacological functions gradually extending from digestive diseases and tumors to metabolic diseases such as obesity, hypertension, and diabetes. The folk proverb “the older, the better,” that is, the longer the storage time, the better the quality, has been mentioned many times in the aging process of *Pericarpium Citri Reticulatae* (orange peel) (8). The data from the present study support that the evolutionary trend of the biological activity of Laoxianghuang appears to follow the same trend as that of *Pericarpium Citri Reticulatae*.

The fermentation of Laoxianghuang from 3 to 20 years can be seen in the fluctuation of its composition. The four substances with more significant changes were α-terpineol, γ-terpinene, cymene, and limonene, of which limonene and its isomer γ-terpinene increased, while the other two decreased. Limonene could be converted to α-terpineol by the action of *Penicillium digitatum* DSM62840 (45) and formed cymene by dehydrogenation (46). Strangely, no corresponding change in its relative content occurred, which needs to be further investigated in our future studies. This may be because, during the fermentation process, Laoxianghuang changes its content and type of components under the action of microorganisms, fermentation additives, and some active factors. Similar to green tea fermented by *Aspergillus niger* PW-2, the α-terpineol content was also significantly reduced (47). In addition, the previous analysis of bacterial diversity by high-throughput sequencing showed that Laoxianghuang contained *Ignatzschineria, Vagococcus, Bifidobacterium*, and *Lactobacillus* (6). Among them, *Bifidobacterium* and *Lactobacillus* could improve the limonene content in cow and goat milk fermentation (48). Unfortunately, the structural characteristics of the microbial community and the evolution of the fermentation process in Laoxianghuang have not been further studied. The categories and contents of the components determine their properties, flavor, and efficacy. However, research on Laoxianghuang is still in the initial exploration and pioneering stage. It is urgent to clarify the biotransformation pathways of the components and the role of microorganisms in the fermentation process.

In summary, an optimized SPME/GC–MS method was developed for the determination of the volatile compounds of Laoxianghuang under the following conditions: 65 μm PDMS/DVB fiber, extraction time of 30 min, and desorption temperature of 200 °C. The composition and relative content of Laoxianghuang fermented for 3, 5, 8, 10, 15, and 20 years were identified under the above conditions, with terpenes and alcohols predominating. The relationship between the category and content of volatile compounds and the year of fermentation was also revealed. Three different stage clusters of Laoxianghuang fermentation were further proposed by chemometrics, and the differential candidate biomarkers were also indicated as α-terpineol, γ-terpinene, cymene, and limonene. Then, through a database search and literature mining, it was clarified that the fermentation process of Laoxianghuang was accompanied by the flavor evolution of citrus from wood to herb, as well as the extension of the effect from digestive aid and anticancer to fat reduction and blood pressure reduction. This study analyzed the changes in the composition, flavor, and function of Laoxianghuang from multiple perspectives and elaborated on its unique flavor and beneficial physiological functions during the fermentation process. This study is expected to provide a useful reference for further research on the fermentation process of Laoxianghuang.

## Data availability statement

The original contributions presented in the study are included in the article/Supplementary material, further inquiries can be directed to the corresponding author.

## Author contributions

LW, LY, and LH designed the study, analyzed, and interpreted the data. XY, LM, YY, and CY collected the samples and entered the data. XY, LM, ZY, HL, YY, and CY performed the laboratory work. LW, LY, and XY wrote the paper. All authors critically reviewed the paper and approved the final version of the paper for submission.

## Funding

This research was funded by the Chaozhou science and technology plan project (Nos. 2020ZX02 and 2020GY02), the Special Innovation Project of General Universities in Guangdong Province (No. 2019KTSCX099), the Key Discipline Project of Guangdong Provincial Education Department (No. 2019-GDXK-0032), and the Guangdong Provincial Key Laboratory of Functional Substances in Medicinal Edible Resources and Healthcare Products (No. 2021B1212040015).

## Conflict of interest

Authors YY and CY were employed by Guangdong Jigong Healthy Food Co., Ltd., Chaozhou, Guangdong, China. The remaining authors declare that the research was conducted in the absence of any commercial or financial relationships that could be construed as a potential conflict of interest.

## Publisher's note

All claims expressed in this article are solely those of the authors and do not necessarily represent those of their affiliated organizations, or those of the publisher, the editors and the reviewers. Any product that may be evaluated in this article, or claim that may be made by its manufacturer, is not guaranteed or endorsed by the publisher.
