# Supplementary material for: SPME-GC–MS combined with chemometrics to assess the impact of fermentation time on the components, flavor, and function of Laoxianghuang
Source: Front Nutr. 2022 Aug 2;9:915776. doi: 10.3389/fnut.2022.915776 (PMC9378830; doi:10.3389/fnut.2022.915776)
Supplement: Supplementary file 1 [file Table_1.DOCX]

Supplementary Material

# Supplementary Table 1. Profile of the compositional features of Laoxianghuang in different fermentation years

|  |  |  |  |  |  | **Relative Content (μg/g)** | | | | | |
| --- | --- | --- | --- | --- | --- | --- | --- | --- | --- | --- | --- |
| **No.** | **RT (min) ^a^** | **CN ^b^** | **CAS** | **MF ^c^** | **Flavor ^d^** | **3^rd^ year** | **5^th^ year** | **8^th^ year** | **10^th^ year** | **15^th^ year** | **20^th^ year** |
| **Terpenes** | | | | | | | | | | | |
| 1 | 5.662 | Thujene | 2867-5-2 | C_10_H_16_ | wood, green, herb | 0 | 0 | 0 | 0.01±0.001 | 0.01±0.001 | 0.01±0.002 |
| 2 | 5.793 | α-Pinene | 7785-26-4 | C_10_H_16_ | pine, turpentine | 0.38±0.026 | 0.43±0.073 | 0.41±0.056 | 0.48±0.042 | 0.72±0.056 | 1.32±0.053 |
| 3 | 6.091 | Camphene | 79-92-5 | C_10_H_16_ | camphor, mint, herb, citrus and green | 0.01±0.001 | 0.04±0.007 | 0.03±0.013 | 0.03±0.003 | 0.06±0.008 | 0.12±0.016 |
| 4 | 6.726 | β-Pinene | 127-91-3 | C_10_H_16_ | pine, resin, turpentine, mint,camphor | 0.01±0.001 | 0.04±0.008 | 0.03±0.020 | 0.08±0.071 | 0.14±0.092 | 0.12±0.019 |
| 5 | 7.012 | Myrcene | 123-35-3 | C_10_H_16_ | balsamic, must, spice,peppery, wood, citrus, mango, mint | 0.31±0.021 | 0.34±0.006 | 0.5±0.032 | 0.56±0.049 | 0.69±0.037 | 1.11±0.213 |
| 6 | 7.384 | α-Phellandrene | 99-83-2 | C_10_H_16_ | turpentine, mint, spice, citrus, herb, peppery | 0 | 0 | 0.04±0.06 | 0.05±0.005 | 0.05±0.007 | 0.1±0.032 |
| 7 | 7.693 | Terpinolene | 586-62-9 | C_10_H_16_ | Pine,citrus,lemon, herb, floral, wood | 5.58±0.381 | 4.14±0.708 | 3.17±0.346 | 2.37±0.207 | 3.13±0.281 | 5.48±0.833 |
| 8 | 7.945 | Cymene | 99-87-6 | C_10_H_14_ | solvent, gasoline, citrus, green pepper, oregano | 13.23±1.902 | 11.2±2.913 | 5.9±0.934 | 5.59±0.489 | 5.21±0.513 | 2.74±0.621 |
| 9 | 8.071 | Limonene | 138-86-3 | C_10_H_16_ | lemon, orange, herb, camphor | 33.64±5.295 | 38.56±6.586 | 39±7.131 | 39.85±5.485 | 43.49±6.823 | 46.15±7.325 |
| 10 | 8.66 | Ocimene | 13877-91-3 | C_10_H_16_ | herb, citrus, floral | 0.09±0.006 | 0.10±0.018 | 0.51±0.384 | 0.63±0.059 | 0.77±0.067 | 1.24±0.357 |
| 11 | 9.055 | γ-Terpinene | 99-85-4 | C_10_H_16_ | gasoline, turpentine, lemon, citrus | 17.11±2.167 | 18.73±3.213 | 21.06±4.328 | 21.64±4.892 | 26.87±5.019 | 28.7±5.216 |
| 12 | 12.162 | Allocimene B | 7216-56-0 | C_10_H_16_ | herb, floral | 0 | 0 | 0.07±0.003 | 0.1±0.009 | 0.1±0.008 | 0.18±0.061 |
| 13 | 22.685 | α-Cubebene | 17699-14-8 | C_15_H_24_ | herb, wax | 0.08±0.005 | 0.08±0.137 | 0.18±0.006 | 0.23±0.020 | 0.15±0.017 | 0.13±0.021 |
| 14 | 23.343 | β-Elemene | 515-13-9 | C_15_H_24_ | herb, wax, fresh | 0 | 0 | 0.03±0.001 | 0.03±0.003 | 0.03±0.002 | 0.03±0.003 |
| 15 | 23.915 | Isocaryophyllene | 118-65-0 | C_15_H_24_ | wood | 0 | 0 | 0.11±0.007 | 0.01±0.001 | 0.01±0.004 | 0.01±0.001 |
| 16 | 24.27 | (E)-α-Bergamotene | 13474-59-4 | C_15_H_24_ | wood, warm, tea | 1.78±0.121 | 3.2±0.556 | 3.04±0.613 | 4.12±0.361 | 1.65±0.163 | 2.12±0.061 |
| 17 | 24.407 | β-Caryophyllene | 87-44-5 | C_15_H_24_ | Lilac, wood, spice, peppery | 0.91±0.062 | 1.12±0.193 | 1.63±0.217 | 2.80±0.249 | 1.20±0.218 | 1.51±0.359 |
| 18 | 25.815 | α-Humulene | 6753-98-6 | C_15_H_24_ | Lilac，wood | 0.06±0.004 | 0.10±0.018 | 0.27±0.064 | 0.26±0.023 | 0.11±0.013 | 0.11±0.020 |
| 19 | 26.032 | (E)-β-Farnesene | 18794-84-8 | C_15_H_24_ | wood, citrus, herb | 0.06±0.005 | 0.07±0.012 | 0.18±0.023 | 0.30±0.027 | 0.09±0.011 | 0.13±0.018 |
| 20 | 26.147 | β-Santalene | 37876-50-9 | C_15_H_24_ | Sandalwood | 0.07±0.005 | 0.14±0.032 | 0.12±0.017 | 0.13±0.019 | 0.13±0.016 | 0.12±0.013 |
| 21 | 26.839 | γ-Muurolene | 30021-74-0 | C_15_H_24_ | herb, wood, spice | 0.17±0.012 | 0.17±0.024 | 1.00±0.281 | 0.50±0.061 | 0.26±0.065 | 0.22±0.067 |
| 22 | 26.999 | Germacrene B | 15423-57-1 | C_15_H_24_ | wood, earth, spice | 0.09±0.006 | 0.10±0.032 | 0.62±0.151 | 0.85±0.384 | 0.41±0.096 | 0.32±0.101 |
| 23 | 27.634 | Isoledene | 29484-27-3 | C_15_H_24_ |  | 0.12±0.008 | 0.08±0.014 | 0.45±0.024 | 0.45±0.073 | 0.24±0.033 | 0.18±0.008 |
| 24 | 27.886 | α-Muurolene | 10208-80-7 | C_15_H_24_ | wood | 0.07±0.005 | 0.07±0.014 | 0.28±0.016 | 0.16±0.021 | 0.09±0.016 | 0.06±0.008 |
| 25 | 28.023 | (Z)-α-Bisabolene | 29837-07-8 | C_15_H_24_ |  | 0.13±0.009 | 0.30±0.052 | 0.31±0.011 | 0.50±0.019 | 0.11±0.008 | 0.14±0.007 |
| 26 | 28.281 | β-Bisabolene | 495-61-4 | C_15_H_24_ | wood, citrusy, floral, fruity，Balsam | 1.65±0.113 | 4.14±0.897 | 4.25±0.391 | 5.28±0.461 | 3.68±0.161 | 2.01±0.203 |
| 27 | 28.47 | γ-Cadinene | 5957-55-1 | C_15_H_24_ | wood | 0.19±0.013 | 0.24±0.041 | 0.55±0.056 | 0.55±0.386 | 0.25±0.073 | 0.12±0.064 |
| 28 | 28.922 | Δ-Cadinene | 483-76-1 | C_15_H_24_ | thyme, herb, wood | 0.44±0.03 | 0.49±0.084 | 1.43±0.147 | 1.32±0.312 | 0.67±0.182 | 0.46±0.135 |
| 29 | 29.82 | 1-Methyl-4-[(2E)-6-Methyl-2,5-Heptadien-2-Yl]Cyclohexene | 25532-79-0 | C_15_H_24_ |  | 0 | 0 | 0.14±0.012 | 0.15±0.053 | 0.05±0.006 | 0.05±0.011 |
| **Alcohols** | | | | | | | | | | | |
| 1 | 11.361 | Fenchol | 1632-73-1 | C_10_H_18_O | camphor, citrus, lime, borneol, pine, lemon,herb,mint | 0.15±0.014 | 0.19±0.032 | 0.23±0.021 | 0.21±0.018 | 0.20±0.021 | 0.08±0.011 |
| 2 | 12.465 | 1-Terpinenol | 586-82-3 | C_10_H_18_O | wood, must | 2.50±0.421 | 0.92±0.157 | 1.38±0.124 | 0.69±0.061 | 0.69±0.071 | 0.23±0.036 |
| 3 | 13.055 | β-Terpinenol | 138-87-4 | C_10_H_18_O | wood, earth | 0.63±0.031 | 0.49±0.084 | 0.38±0.035 | 0.21±0.018 | 0.17±0.017 | 0.08±0.011 |
| 4 | 13.776 | DL-Isoborneol | 124-76-5 | C_10_H_18_O | must, camphor,herb, mint,wood | 0.08±0.005 | 0.05±0.008 | 0.05±0.005 | 0.03±0.003 | 0.03±0.003 | 0.02±0.002 |
| 5 | 14.371 | Borneol | 464-45-9 | C_10_H_18_O | camphor, pine, wood, earth, mint, herb | 0.30±0.026 | 0.29±0.049 | 0.28±0.025 | 0.23±0.021 | 0.24±0.025 | 0.1±0.014 |
| 6 | 14.892 | (+)-Menthol | 15356-60-2 | C_10_H_20_O | mint | 0 | 0 | 0.17±0.016 | 0.01±0.001 | 0.02±0.002 | 0.02±0.003 |
| 7 | 15.2 | Terpinen-4-ol | 562-74-3 | C_10_H_18_O | turpentine, nutmeg, must, pepper, wood, citrus | 0.42±0.029 | 0.21±0.036 | 1.36±0.122 | 0.53±0.047 | 0.39±0.039 | 0.11±0.015 |
| 8 | 16.133 | α-Terpineol | 98-55-5 | C_10_H_18_O | Lilac, oil, anise, mint, pine, citrus, wood, lemon, soap | 13.15±4.321 | 10.22±3.746 | 7.75±1.696 | 5.14±0.949 | 4.54±0.461 | 1.89±0.267 |
| 9 | 16.476 | γ-Terpineol | 8000-41-7 | C_10_H_18_O | wood, pine, floral, lime | 2.17±0.436 | 1.07±0.149 | 1.36±0.097 | 0.77±0.088 | 0.69±0.093 | 0.29±0.062 |
| 10 | 23.074 | Nerol | 106-25-2 | C_10_H_18_O | sweet, neroli, citrus, magnolia, lemon | 0 | 0 | 0.10±0.210 | 0.18±0.268 | 0.05±0.006 | 0.02±0.004 |
| **Aldehydes** | | | | | | | | | | | |
| 1 | 4.02 | 2-Furaldehyde | 13529-27-6 | C_5_H_4_O_2_ |  | 1.03±0.219 | 0.46±0.138 | 0.09±0.021 | 0.08±0.014 | 0.06±0.008 | 0.01±0.002 |
| 2 | 6.457 | 5-Methylfurfural | 620-02-0 | C_6_H_6_O_2_ | almond, caramel, burnt sugar, spice, maple, grain, maple | 0.12±0.037 | 0.03±0.004 | 0.01±0.001 | 0.01±0.001 | 0.01±0.001 | 0.01±0.003 |
| 3 | 10.909 | 1-Nonanal | 124-19-6 | C_9_H_18_O | fat, citrus, green, rose, orange, cucumber, melon, potato, nutty, coconut | 0.06±0.006 | 0.04±0.006 | 0 | 0 | 0 | 0 |
| **Ketones** | | | | | | | | | | | |
|  | 5.078 | Cyclohexanone | 108-94-1 | C_6_H_10_O | mint | 0.24±0.014 | 0.31±0.067 | 0.02±0.007 | 0.01±0.001 | 0.01±0.001 | 0 |
|  | 15.761 | 3-Methyl Acetophenone | 585-74-0 | C_9_H_10_O |  | 0 | 0 | 0.03±0.008 | 0.08±0.013 | 0.05±0.009 | 0.03±0.006 |
|  | 18.084 | D(+)-Carvone | 2244-16-8 | C_10_H_14_O | spice, mint, bread, caraway | 0.02±0.003 | 0.03±0.008 | 0.10±0.086 | 0.17±0.068 | 0.14±0.071 | 0.02±0.043 |
|  | 27.308 | β-Ionone | 79-77-6 | C_13_H_20_O | seaweed, violet, flower, raspberry, wood, orrisl, berry | 0 | 0 | 0.05±0.009 | 0.03±0.048 | 0.03±0.053 | 0.03±0.062 |
| **Ethers** | | | | | | | | | | | |
| 1 | 35.983 | N-Octyl Ether | 629-82-3 | C_16_H_34_O |  | 0.55±0.062 | 0.08±0.007 | 0.10±0.013 | 0.01±0.002 | 0.01±0.001 | 0.01±0.001 |
| 2 | 19.412 | Anethole | 4180-23-8 | C_10_H_12_O | sweet, anise, licorice, mimosa | 0.02±0.002 | 0.02±0.003 | 0.04±0.007 | 0.10±0.067 | 0.06±0.008 | 0.09±0.011 |
| **Esters** | | | | | | | | | | | |
| 1 | 17.587 | Bornyl Formate | 7492-41-3 | C_11_H_18_O_2_ | green, earth, herb, balsam, pine | 0 | 0 | 0.03±0.006 | 0.04±0.004 | 0.03±0.005 | 0.03±0.005 |
| 2 | 21.867 | Citronellyl Acetate | 150-84-5 | C_12_H_22_O_2_ | rose, dust, floral, green, citrus, wood, fruit, wax, aldehydic, pear, apple | 0 | 0.01±0.001 | 0.05±0.007 | 0.07±0.006 | 0.02±0.001 | 0.01±0.001 |
| 3 | 22.313 | Neryl Acetate | 141-12-8 | C_12_H_20_O_2_ | fruit, floral, rose, soap, citrus, dewy, pear | 0 | 0 | 0.19±0.034 | 0.15±0.048 | 0.05±0.007 | 0.03±0.004 |
| **Phenols** | | | | | | | | | | | |
| 1 | 19.526 | Thymol | 89-83-8 | C_10_H_14_O | herb, thyme, camphor, wood, spicy | 0.13±0.059 | 0.68±0.068 | 0.89±0.054 | 0.50±0.037 | 0.37±0.022 | 0.23±0.028 |
| 2 | 21.094 | Chlorothymol | 89-68-9 | C_10_H_13_ClO |  | 0.06±0.005 | 0.03±0.007 | 0.03±0.006 | 0.03±0.011 | 0.03±0.007 | 0.01±0.001 |
| 3 | 22.073 | Eugenol | 97-53-0 | C_10_H_12_O_2_ | sweet, spicy, clove, wood | 0.02±0.005 | 0.03±0.006 | 0.12±0.048 | 0.15±0.051 | 0.11±0.046 | 0.12±0.038 |
| **Others** | | | | | | | | | | | |
| 1 | 5.021 | Furan, 2,4-dimethyl- | 3710-43-8 | C_6_H_8_O |  | 0.04±0.003 | 0.11±0.065 | 0 | 0 | 0 | 0 |

^a^ RT: Retention Time.

^b^ CN: Component Name

^c^ MF:Molecular Formula

^d^ Flavor queried from Flavornet (http://www.flavornet.org/flavornet.html) and Odour Database (http://www.odour.org.uk/odour/index.html).
